# Application of Shannon Entropy to Reaction–Diffusion Problems Using the Stochastic Finite Difference Method

**DOI:** 10.3390/e27070705

**Published:** 2025-06-30

**Authors:** Marcin Kamiński, Rafał Leszek Ossowski

**Affiliations:** Faculty of Civil Engineering, Architecture and Environmental Engineering, Lodz University of Technology, 93-590 Łódź, Poland; rafal.ossowski@p.lodz.pl

**Keywords:** reaction–diffusion problem, Shannon entropy, computer symbolic analysis, stochastic finite difference method, generalized stochastic perturbation technique, time series

## Abstract

In this study, we introduce Shannon entropy as a key metric for assessing concentration variability in diffusion processes. Shannon entropy quantifies the uncertainty or disorder in the spatial distribution of diffusing particles, providing a novel perspective on diffusion dynamics. This proposed approach enables a more comprehensive characterization of mixing efficiency, equilibrium states, and transient diffusion behavior. Numerical simulations performed using the finite difference method in the MAPLE 2025 symbolic computing environment illustrate how entropy evolution correlates with diffusion kinetics. The computational model used in this study is based on a previously developed framework from our earlier research, ensuring consistency and validation of the results. The findings suggest that Shannon entropy can serve as a robust descriptor of diffusion-driven mixing, with potential applications in engineering, environmental science, and biophysics.

## 1. Introduction

The finite difference method (FDM) [1,2] is a fundamental numerical technique used in computational engineering to approximate the solutions of differential equations. It has been widely applied in fields such as heat transfer [3], electromagnetics [4], and geomechanics, where partial differential equations (PDEs) often arise. The classical FDM approach replaces continuous derivatives with discrete finite difference approximation, offering a straightforward and efficient tool for solving boundary value problems on structured grids [5]. In recent years, research has increasingly focused on extending the FDM to stochastic settings, where system parameters or boundary conditions exhibit randomness. This leads to implementation and application of the stochastic finite difference method (SFDM), which enables the analysis of uncertainty propagation in systems modeled by PDEs. A prominent approach within this context is the generalized stochastic perturbation technique [6,7], which involves Taylor series expansions of the stochastic input variables. This method has previously been applied in the context of the finite element method (FEM) [6], and the boundary element method (BEM) [7], and here we adapt it for the FDM with novel capabilities: (1) arbitrary-order stochastic expansions, (2) compatibility with general probability distributions [8], and (3) symbolic computation of hierarchical equations using the MAPLE 2025 system. The stochastic perturbation method is frequently contrasted with the Karhunen-Loeve and polynomial chaos expansion [9] or the Monte-Carlo simulation approach [10,11]. In this study, we apply the SFDM to a reaction–diffusion problem—a class of models that describe how chemical substances diffuse and react over time and space. Reaction–diffusion equations close to the advection-diffusion ones [12] are central in various scientific disciplines, including biophysics [13], chemical engineering, and ecology, due to their ability to capture both spatial transport and reaction dynamics. Our implementation focuses on one-dimensional steady and unsteady cases with a single uncertain parameter, although the methodology is general and can be extended to multi-dimensional and nonlinear problems.

A key contribution of this work is the introduction of Shannon (information) entropy as a metric for quantifying uncertainty in concentration profiles over time [14]; such type of the entropy has been used also in structural damage identification [15]. While classical diffusion analysis often relies solely on concentration gradients or variances, entropy provides a complementary perspective, capturing disorder and mixing efficiency in a probabilistic sense [16,17]. By tracking the temporal evolution of entropy in stochastic diffusion processes, we gain insight into system behavior, equilibrium tendencies, and the influence of input variability [18,19]. Although entropy-based methods have been used in theoretical studies of diffusion [20,21,22], their integration into computational frameworks for uncertainty quantification is still relatively rare [23,24]. Let us note that fractional diffusion and fractional order derivatives became recently very popular in mathematical and numerical modeling [25,26]. 

Our work addresses this gap by embedding entropy computation directly into the SFDM algorithm. We present numerical experiments that demonstrate how entropy evolves in time and space, reflecting the interplay between diffusion kinetics and stochastic variability. The results are verified using symbolic differentiation and parametric studies, with particular attention to the role of the input uncertainty [27]. In summary, this study offers a computational framework that combines stochastic finite difference modeling with entropy-based uncertainty analysis. This approach enables a more comprehensive understanding of diffusion-driven processes and provides a foundation for future extensions to complex, real-world systems with uncertain inputs.

## 2. Deterministic Model

**Figure 1:**
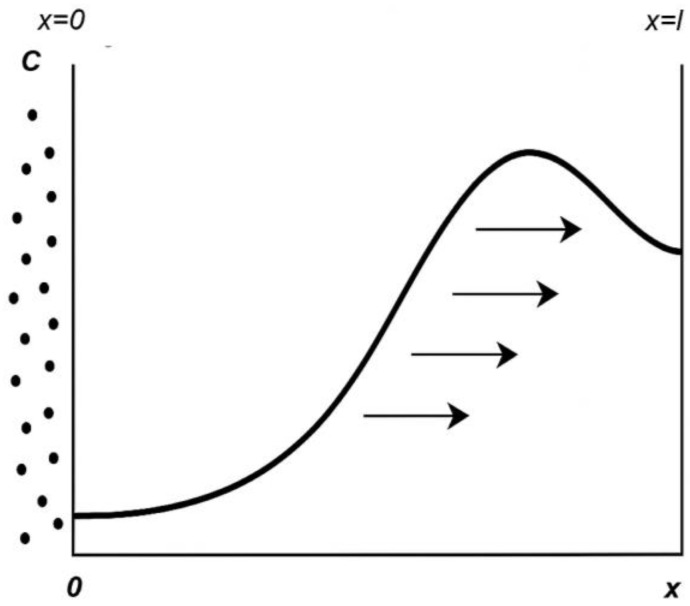
Simple 1D idealization of the diffusion process (concentration C(x) of the molecules).

We begin by formulating a deterministic boundary value problem describing the steady-state concentration profile C(x), where x∈R, within a classical reaction–diffusion framework. This formulation applies to a linear system governed by reaction–diffusion dynamics over a one-dimensional spatial domain, as discussed in the literature [28,29]. This scenario assumes a planar region in which molecular concentration exhibits spatial variability, while the boundary conditions and model parameters remain fixed and deterministic. A conceptual representation of the concentration field within this domain is provided in Figure 1—although presented schematically, the analysis and numerical model refer strictly to a one-dimensional domain.

This problem is described by a system of differential equations:

(1) $$\begin{array}{c} \frac{d^{2}C(x)}{dx^{2}}-C(x)=0,\\ {C\left(x\right)|}_{x=0}=1,\\ {\frac{dC(x)}{dx}|}_{x=l}=0, \end{array}$$

for which a closed-form analytical solution is available and given by $C_{an}(x)=\frac{2e\cdot \cosh\left(x-1\right)}{1+e^{2}}$, where *e* denotes Euler’s number, x denotes the current coordinate, and *l* stands for the channel length. This closed-form expression serves as a benchmark for validating the numerical accuracy of the proposed finite difference scheme, particularly in the context of preparing the computational framework for entropy-based uncertainty quantification. Alternatively, the same problem can be approached numerically using the finite difference method (FDM). In this approach, the spatial domain [0,*l*] is discretized into a finite number of the uniform subintervals of length Δ, where the nodal positions are defined as $x_{i}=\left(i-1\right)\cdot ∆$, for i=1,…,n+1. The approximations of concentration values at these discrete locations are denoted by $C_{i}$. Spatial derivatives appearing in the governing equations are approximated using central difference schemes, as detailed in [5]:

(2) $${\left(\frac{dC(x)}{dx}\right)}_{i}=\frac{{C(x)}_{i+1}-{C(x)}_{i-1}}{2\Delta },$$

(3) $${\left(\frac{d^{2}C(x)}{dx^{2}}\right)}_{i}=\frac{{C(x)}_{i+1}-2{C(x)}_{i}+{C(x)}_{i-1}}{\Delta^{2}}.$$

The further numerical solution needs a definition of the imposed boundary conditions, namely

(4) $${C(x)}_{1}=1,$$

(5) $$\frac{{C(x)}_{n+2}-{C(x)}_{n}}{2\Delta }=0,$$

for *x_n+_*_2_ being a fictitious node out of the initial domain. Then, we solve for $C_{i}$, i=1,…,n from the following linear equations system:

(6) $$\begin{array}{c} {C(x)}_{1}=1,\\ {C(x)}_{3}-\left(2+\Delta^{2}\right){C(x)}_{2}+{C(x)}_{1}=0,\\ ...\\ {C(x)}_{n+2}-\left(2+\Delta^{2}\right){C(x)}_{n+1}+{C(x)}_{n}=0,\\ {C(x)}_{n+2}-{C(x)}_{n}=0, \end{array}$$

which can be represented as the matrix equation

(7) KC=F

with tri-diagonal coefficients:

(8) $$K=\left(\begin{array}{cccccc} 1 & 0 & 0 & 0 & ... & 0\\ 1 & -(2+\Delta^{2}) & 1 & 0 & ... & 0\\ 0 & 1 & -(2+\Delta^{2}) & 1 & ... & 0\\ ... & ... & ... & ... & ... & ...\\ 0 & 0 & 0 & 1 & -(2+\Delta^{2}) & 1\\ 0 & 0 & 0 & -1 & 0 & 1 \end{array}\right),\;F=\left(\begin{array}{c} 1\\ 0\\ 0\\ ...\\ 0\\ 0 \end{array}\right).$$

As is known, the numerical solution to this problem by the FDM has quadratic convergence since the discretization errors for both first- and second-order derivatives are equal to $O(\Delta^{2})$. The solution to the non-linear boundary value problem is equivalent to the reaction–diffusion problem [28]:

(9) $$\frac{d^{2}C(x)}{dx^{2}}=\alpha C\left(x\right)+x^{2}+\beta C^{3}\left(x\right),$$

with slightly modified boundary conditions introduced to test the sensitivity of the stochastic solution

(10) $$C\left(0\right)=0=C\left(1\right).$$

One may then proceed with the discretization n×∆=l in quite a similar way until one obtains

(11) $$\begin{array}{c} {C(x)}_{1}=0,\\ {C(x)}_{i+1}-2{C(x)}_{i}+{C(x)}_{i-1}-\Delta^{2}\left(\alpha {C(x)}_{i}+(i-1{)}^{2}\Delta^{2}+\beta C_{i}^{3}(x)\right)=0,\\ {C(x)}_{n+1}=0, \end{array}\;i=2,\ldots ,n.\;$$

As we will see in the next section, we can also provide the multipoint versions of the finite difference method for the needs of the stochastic computational solution or apply some error analysis techniques to determine the accuracy of determination of the particular probabilistic moments [30].

**Figure 2:**
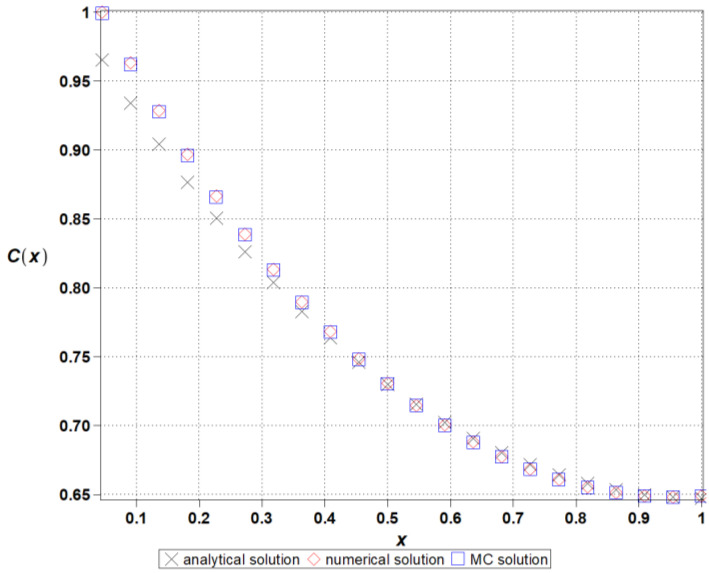
The concentration field *C*[*x*] over the domain xϵ[0,…,l] validation of the proposed stochastic finite difference method by comparison with the reference (analytical) solution for the reaction–diffusion system.

Figure 2 presents the numerical validation of the finite difference method (FDM) applied to the reaction–diffusion system. The computed solution is compared with the reference deterministic profile, demonstrating good consistency across the spatial domain. The observed agreement confirms the numerical stability and accuracy of the implemented scheme, providing a reliable foundation for the subsequent entropy-based uncertainty quantification. Ensuring fidelity at this stage is essential, as even minor numerical artifacts could distort entropy metrics in stochastic extensions.

## 3. A Stochastic Process Approach

The theory of stochastic processes provides a mathematical framework for describing systems that evolve over time and space according to probabilistic laws. This formalism is extensively applied in diverse fields of science and engineering to model complex, inherently uncertain phenomena [31]. In the context of reaction–diffusion problems, we now extend the formulation from a random variable $b\left(\omega \right)$, which induces a spatially random field $C\left(x,\omega \right)$, to a time-dependent random variable $b\left(\omega ,t\right)$, leading to a spatiotemporal stochastic field $C\left(x,\omega ,t\right)$. Let us consider a probability space $\left(\Omega ,\;S,\;P\right)$, and a discrete time domain T⊂R. A function X:T×Ω→R is defined as a stochastic process if the following condition holds:

(12) $$\wedge_{t\in T}\wedge_{x\in R}\left\{\omega :X(t,\omega )<x\right\}\in S,$$

which means that for each fixed time t, the function $X\left(t,\;\cdot \right)$ is a random variable on $\left(\Omega ,\;S,\;P\right)$. In this framework, the concentration field C can be expressed as a function of space, probability, and time:

(13) $$C(x,\omega ,t)=C_{t}(x,\omega ).$$

Analogously to classical random variables, stochastic processes are characterized by statistical descriptors such as the expectation, variance, and higher-order moments [31]:

- expected value

(14) $$E\left(X_{t}\right)=m\left(t\right)=m\left(t+\tau \right),\forall_{\tau }\in R;$$

- variance

(15) $$Var\left(X_{t}\right)=\sigma^{2}\left(X_{t}\right)=E\left((X_{t}-E[X_{t}]{)}^{2}\right);$$

- autocovariance

(16) $$K\left(X_{t_{1}},X_{t_{2}}\right)=E\left(\left(X_{t_{1}}-E\left[X_{t_{1}}\right]\right)\left(X_{t_{2}}-E\left[X_{t_{2}}\right]\right)\right);$$

- autocorrelation

(17) $$R\left(X_{t_{1}},X_{t_{2}}\right)=E\left(X_{t_{1}}\cdot X_{t_{2}}\right);$$

- autocorrelation coefficient

(18) $$\rho (X_{t_{1}},X_{t_{2}})=\frac{K\left(X_{t_{1}},X_{t_{2}}\right)}{StD(X_{t_{1}})StD(X_{t_{2}})}.$$

We consider a specific example of a stochastic process given by a power-type time series of the form

(19) $$X_{t}=A\cdot t^{n},\;\;t\in R,n\in N.$$

where A is a Gaussian (normally distributed) random variable. This formulation introduces randomness through the multiplicative coefficient, while the time dependence follows a deterministic power-law structure. It should be noted that the use of a polynomial time series (19) does not limit the presented method solely to the analysis of polynomial entropy.

Based on this definition, the key central probabilistic moments of the process—namely, the expectation, variance, and the *k*th central moment—can be derived analytically:

(20) $$m(X_{t})=E(X_{t})=\int_{-\infty }^{+\infty }x(t)p(x)dx=E[A]\cdot t^{n},$$

(21) $$Var(X_{t})=\int_{-\infty }^{+\infty }{\left(x(t)-E(X_{t})\right)}^{2}p(x)dx=Var(A)\cdot t^{2n},$$

(22) $$\mu_{k}(X_{t})=\int_{-\infty }^{+\infty }{\left(x(t)-E(X_{t})\right)}^{k}p(x)dx=\mu_{k}(A)\cdot t^{n\cdot k}.$$

The computational algorithm including the time series in the SFDM analysis using the generalized stochastic perturbation technique described before for a single random variable is displayed using the flowchart in Table 1.

The core concept, illustrated schematically in Table 1, involves discretizing the temporal domain using a uniform time step Δt. At each discrete time point, the first two probabilistic moments (i.e., the mean and variance) of the input stochastic time series describing the channel height are computed. Subsequently, the boundary value problem is solved iteratively using the response function method (RFM) integrated with the stochastic finite difference method. In this approach, the stochastic response function is approximated by a low-order polynomial expansion in terms of the input random variable, and the coefficients of this expansion are determined using symbolic or numerical techniques. This allows efficient estimation of output probabilistic moments by analytical integration over the input probability density function. The RFM-based framework enables a parametric analysis of the system behavior, capturing the dependence of the diffusion process on both time and the variability of uncertain parameters, while significantly reducing the computational cost compared to traditional Monte Carlo simulations.

All the computations are completed with iterative determination of the Shannon entropy [14,18,19], which is presented as a function of time during the diffusion process. An explanation of the final point in this algorithm is delivered for a continuous random variable *b* having the probability density function $g_{b}\left(x\right)$ discretized by a set of subintervals of the same length *δ*; this formulation directly follows Monte Carlo simulation-based histograms. The following representation of $g_{b}\left(x\right)$ can be made in each *i*th subinterval [24]:

(23) $$g_{b}\left(x_{i}\right)=\frac{1}{\delta }\int_{i\delta }^{\left(i+1\right)\delta }g_{b}\left(x\right)\;dx.$$

Further, let us introduce the additional quantized random variable $\hat{x}$ defined as

(24) $$\hat{x}=x_{i}\;for\;i\delta \leq \hat{x}\leq \left(i+1\right)\delta ,$$

whose probability equals

(25) $$p_{i}=\int_{i\delta }^{\left(i+1\right)\delta }g_{b}\left(x\right)dx=\delta g_{b}\left(x_{i}\right).$$

So, probabilistic entropy according to the Shannon definition corresponding to a partition of the histogram of the density function $g_{b}\left(x\right)$ into *n* equal subsets can be calculated as

(26) $$h\left(\hat{x}\right)=-\sum_{i=1}^{n}p_{i}\log\left(p_{i}\right)=-\sum_{i=1}^{n}\delta g_{b}\left(x_{i}\right)\log\left(\delta g_{b}\left(x_{i}\right)\right)=\;\;-\sum_{i=1}^{n}\delta g_{b}(x_{i})\log\left(g_{b}(x_{i})\right)-\log\left(\delta \right).$$

Symbol *h* refers here to the *h*-theorem proposed by Boltzmann and is further used to traditionally denote probabilistic entropy under consideration.

## 4. Reaction–Diffusion with Uncertainty

We now consider the following boundary value problem defined on a stochastic domain:

(27) $$\begin{array}{c} \frac{d^{2}C(x,\omega ,t)}{dx^{2}}-C\left(x,\omega ,t\right)=0,\;\;x\epsilon \left(0,b\left(\omega \right)\right),\;\;\;\;\;\;\;\omega \epsilon \Omega ,t\in \left[0,T\right]\;\\ {C\left(x,\omega ,t\right)|}_{x=0}=1,\;\;\;\;\;\;\;\;\;\;\;\;\;\;\;\;\;\;\;\;\;\;\;\;\;\;\;\;\;\;\;\;\;\;\;\;\;\;\;\;\;\;\;\;\;\;\;\;\;\;\;\;\omega \epsilon \Omega ,t\in \left[0,T\right]\\ {\frac{dC(x,\omega ,t)}{dx}|}_{x=b\left(\omega \right)}=0,\;\;\;\;\;\;\;\;\;\;\;\;\;\;\;\;\;\;\;\;\;\;\;\;\;\;\;\;\;\;\;\;\;\;\;\;\;\;\;\;\;\;\;\;\;\omega \epsilon \Omega ,t\in \left[0,T\right] \end{array}$$

where $b\left(\omega \right)\in R_{+}$ is a truncated Gaussian random variable. The concentration $C\left(x,\omega ,t\right)$ is modeled as a polynomial time series whose coefficients are also truncated Gaussian variables with prescribed first and second probabilistic moments. To solve this stochastic boundary value problem, the generalized stochastic perturbation method is employed. In this framework, the random variable *b* is characterized by a known probability density function $p\left(b\right)$, and the stochastic response function $F\left(b,t\right)$ is analyzed in terms of its central probabilistic moments. The mth central moment is defined as

(28) $$\mu_{m}\left(F(b,t)\right)=\int_{-\infty }^{+\infty }{\left(F(b,t)-E[F(b,t)]\right)}^{m}p(b)db.$$

The fundamental idea behind the stochastic perturbation approach is inspired by classical Taylor expansions. Both input variables and state functions are approximated using truncated Taylor series expanded about their mean values. The expansion is formulated in terms of a small perturbation parameter ε>0. For instance, the *n*th-order truncated expansion of a stochastic concentration function $C\left(b,t\right)$ can be written as [27]

(29) $$C\left(b,t\right)=C^{0}\left(b^{0},t\right)+\sum_{k=1}^{n}\frac{\varepsilon^{k}}{k!}{\left(\Delta b\right)}^{k}\frac{\partial^{k}C(b,t)}{\partial b^{k}},$$

where

(30) $$\varepsilon \Delta b=\varepsilon \left(b-b^{0}\right)$$

is the first variation of *b* about its expected value and, similarly,

(31) $$\varepsilon^{2}{\left(\Delta b\right)}^{2}=\varepsilon^{2}{\left(b-b^{0}\right)}^{2}$$

is the second variation of *b* around its expected value, where *n*th-order variation can be expressed accordingly. Traditionally, the stochastic perturbation approach to all the physical problems is entered by the respective perturbed equations of the *zero*th, first, and successively higher orders being a modification of the initial deterministic formulation. There holds for $\tau \epsilon \left[0,\infty )\right.$:

- the zeroth order partial differential equation

(32) $$\frac{d^{2}C^{0}(\tau )}{dx^{2}}-C^{0}\left(\tau \right)=0;$$

- the *n*th-order partial differential equation

(33) $$\frac{d^{2}}{dx^{2}}\left(\frac{\partial^{n}C(\tau )}{\partial b^{n}}\right)-\frac{\partial^{n}C\left(\tau \right)}{\partial b^{n}}=0.$$

Having solved those equations for $C^{0}\left(\tau \right)=C^{0}\left(x,\tau \right)$ and its higher orders, respectively, (specifically its partial derivatives with respect to random input within all discrete points of the grid), we derive the expressions for the expected values and the other moments. Since we propose the finite difference scheme to solve these equations numerically, we need to expand additionally Equation (7) to obtain the stochastic finite difference method scheme. There holds for $\tau \epsilon \left[0,\infty )\right.$:

- the zeroth equation

(34) $$K^{0}\left(\tau \right)C^{0}\left(\tau \right)=F^{0}\left(\tau \right);$$

- the linear recursive equation for each next perturbation order *k*

(35) $$\sum_{i=1}^{k}\left(\begin{array}{c} k\\ i \end{array}\right)\frac{\partial^{i}K(\tau )}{\partial b^{i}}\frac{\partial^{k-i}C(\tau )}{\partial b^{k-i}}=\frac{\partial^{k}F(\tau )}{\partial b^{k}}$$

to be solved all consecutively to determine the probabilistic characteristics of the random output. In order to calculate the expected values and higher-order probabilistic moments of $C\left(x,\omega ,t\right)$, the same Taylor expansion is employed for the definitions of its probabilistic moments like

(36) $$E\left[C\left(b,t\right)\right]=\int_{-\infty }^{+\infty }C(b,t)p(b)db=\int_{-\infty }^{+\infty }\left(C^{0}\left(b^{0},t\right)+\sum_{k=1}^{n}\frac{\varepsilon^{k}}{k!}{\left(\Delta b\right)}^{k}\frac{\partial^{k}C\left(b,t\right)}{\partial b^{k}}\right)p\left(b\right)db.$$

If there is a high random dispersion in the input random variable and the symmetric probability density function is chosen, then the generalized expansion simplifies to

(37) $$E\left[C\left(b,t\right)\right]=C^{0}\left(b^{0},t\right)+\sum_{k=1}^{n}\frac{\varepsilon^{2k}}{\left(2k\right)!}\frac{\partial^{2k}C\left(b,t\right)}{\partial b^{2k}}\mu_{2k}\left(b\right),$$

where $\mu_{2k}(b)$ denotes the *2k*th order probabilistic moment of the variable b. When the probability density function is defined as the Gaussian with the standard deviation *σ*(*b*), we additionally obtain

(38) $$\mu_{2k+1}\left(b\right)=0,\;\;\;\mu_{2k}\left(b\right)=\left(2k-1\right)!!\sigma^{2k}\left(b\right).$$

Using such an extension of the random input, a desired efficiency of the expected values can be achieved by the appropriate choice of the perturbation parameter and maximum order corresponding to the particular input probability density function type, probabilistic moments interrelations, acceptable error of the computations, etc. This choice can reasonably be made by the comparative studies with the Monte Carlo simulations or theoretical results obtained by the direct (i.e., symbolic) integration. Consequently, the *m*th-order probabilistic moment of the system response is evaluated based on the polynomial representation constructed using the response function method. In this approach, the system response is approximated by a response function—typically a low-degree polynomial in the input random variable—whose coefficients are obtained through symbolic or numerical procedures. This method enables analytical integration with respect to the probability density function of the input parameter, yielding closed-form expressions for expectations, variances, and higher-order moments:

(39) $$\mu_{m}\left(C(b,t)\right)=\int_{-\infty }^{+\infty }{\left(\left(C^{0}\left(b^{0},t\right)+\sum_{k=1}^{n}\frac{\varepsilon^{k}}{k!}{\left(\Delta b\right)}^{k}\frac{\partial^{k}C(b,t)}{\partial b^{k}}\right)-E[C(b,t)]\right)}^{m}p(b)db=\;\int_{-\infty }^{+\infty }{\left(\sum_{k=1}^{n}\frac{\varepsilon^{k}}{k!}{\left(\Delta b\right)}^{k}\frac{\partial^{k}C(b,t)}{\partial b^{k}}\right)}^{m}p(b)db.$$

The computational effort remains comparable to the deterministic case and avoids the high cost of large-scale Monte Carlo simulations, which require a substantial number of samples and postprocessing steps such as regression via the least squares method. Let us also note that the expansion provided in Equation (39) may be completed automatically in computer algebra systems for the given natural order.

## 5. Numerical Illustration

**Figure 3:**
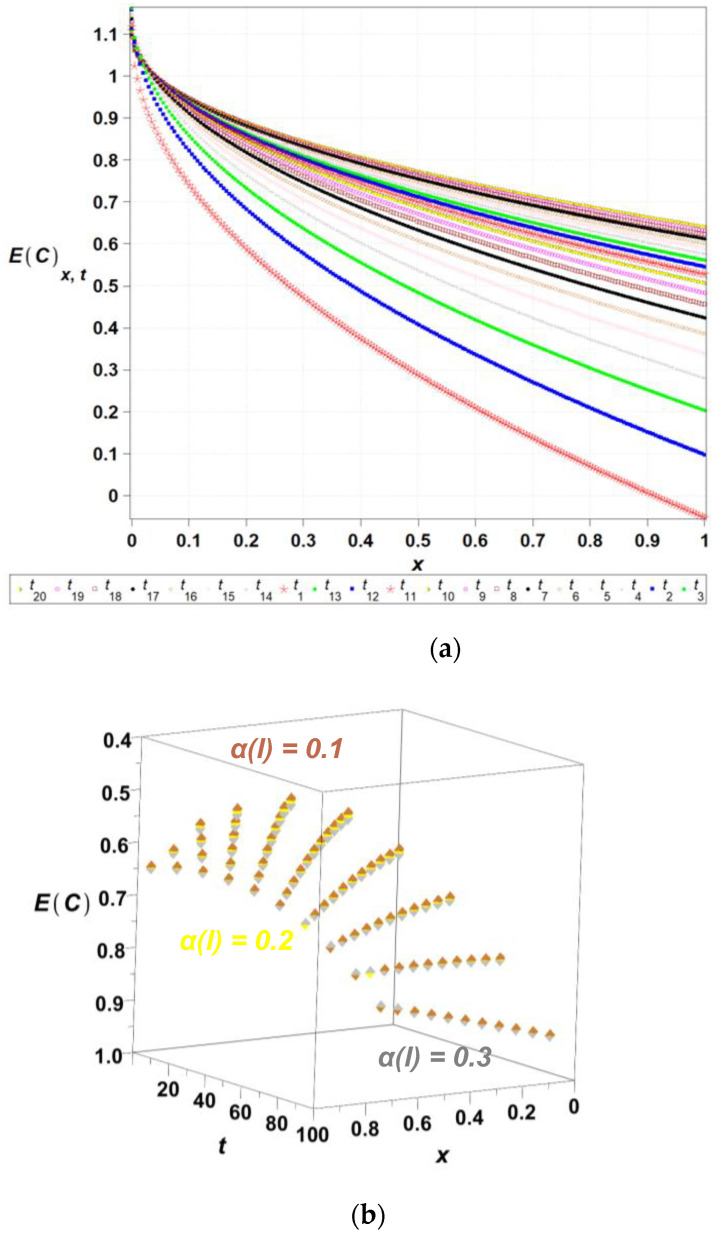
The expected values of the concentration *E*(*C*[*x*,*t*]) *for selected time* $t_{i},i=\{1,2,\ldots ,20\}$, over the domain xϵ[0,…,1] (**a**) and 3D plot of the expected concentration field *E*(*C*[*x*,*t*]) for spatially varying diffusion coefficients ∝(l)ϵ{0.1,0.2,0.3}, tϵ[0,…,100] (**b**).

**Figure 4:**
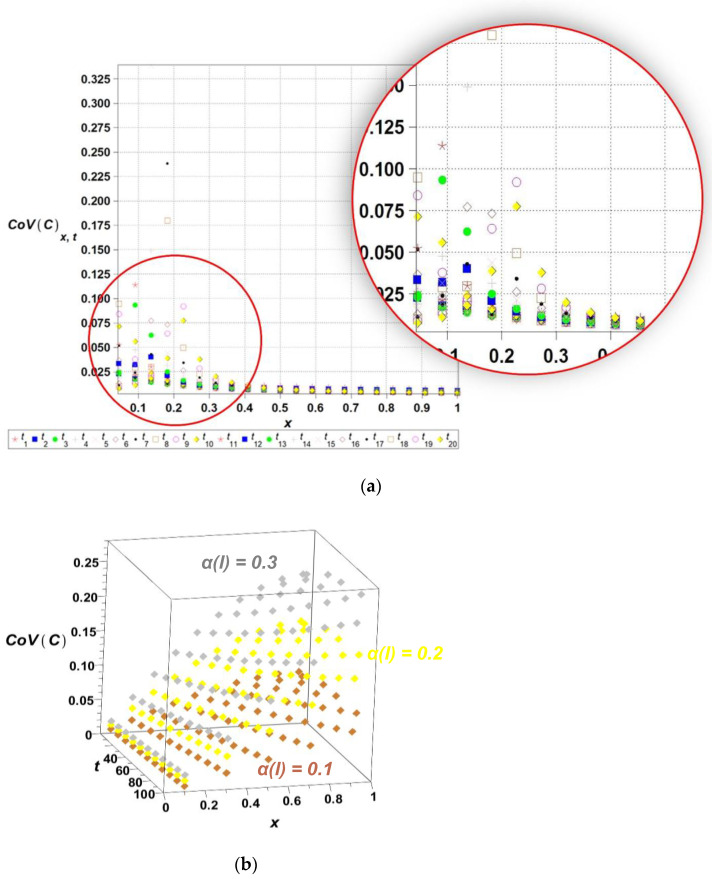
The coefficients of variation of the concentration *CoV*(*C*[*x*,*t*]) for selected time $t_{i},i=\{1,2,\ldots ,20\}$, over the domain xϵ[0,…,1] (**a**) and 3D plot of the coefficients of variation of the concentration *CoV*(*C*[*x*,*t*]) for spatially varying diffusion coefficients ∝(l)ϵ{0.1,0.2,0.3}, $t\epsilon \left[0,\ldots ,100\right]\;$(**b**).

**Figure 5:**
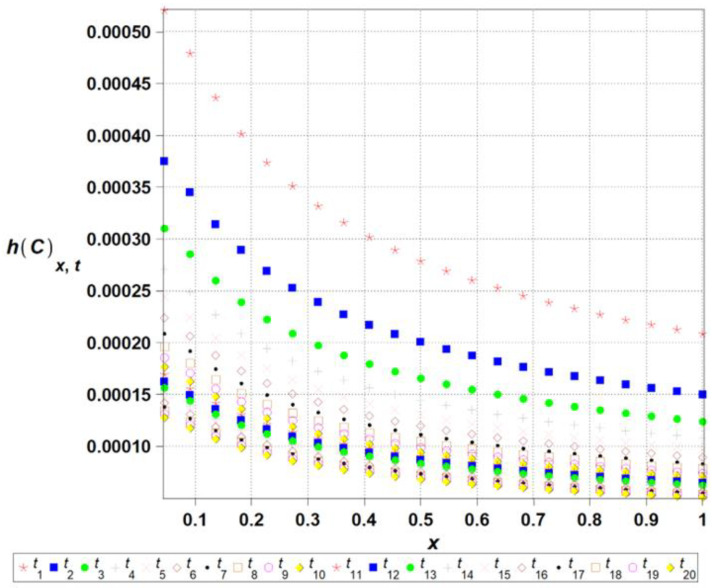
The entropy of the concentration *h*(*C*[*x*,*t*]) for selected time $t_{i},i=\{1,2,...,20\}$, over the domain xϵ[0,...,1].

**Figure 6:**
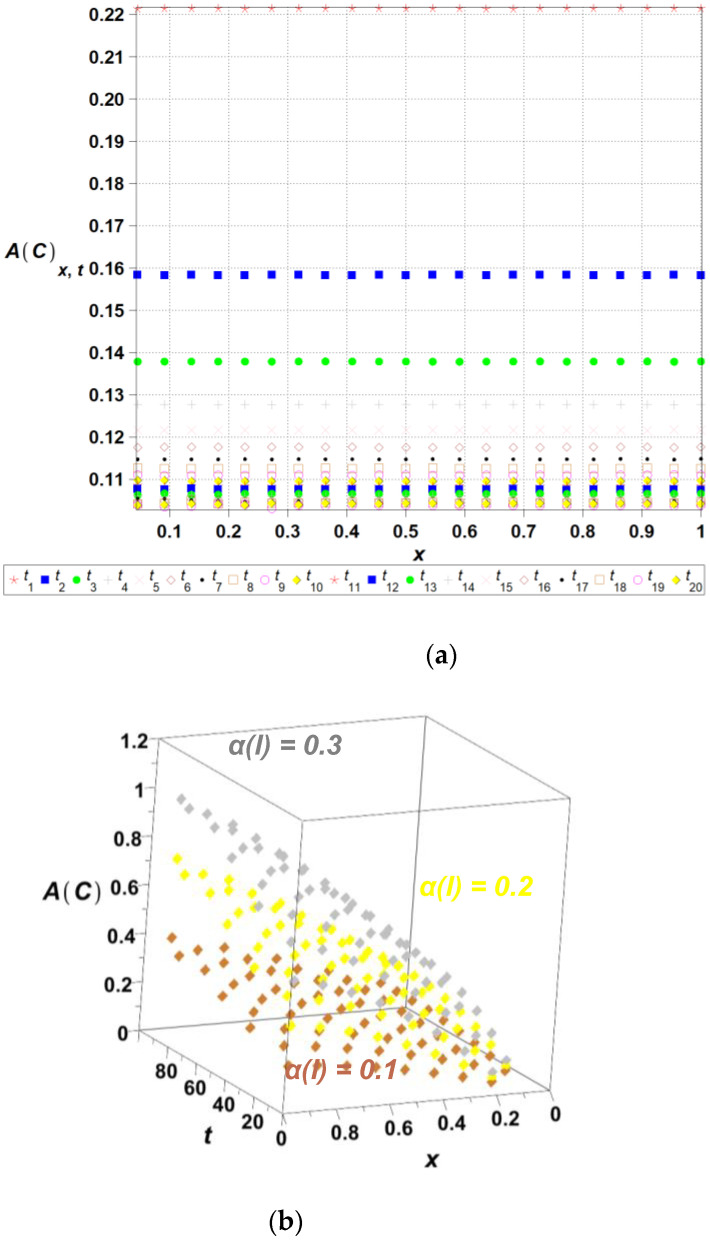
The skewness of concentration *A*(*C*[*x*,*t*]) for selected time $t_{i},i=\{1,2,\ldots ,20\}$, over the domain xϵ[0,...,1] (**a**) and 3D plot of the skewness of concentration *A*(*C*[*x*,*t*]) for spatially varying diffusion coefficients ∝(l)ϵ{0.1,0.2,0.3}, tϵ[0,…,100] (**b**).

**Figure 7:**
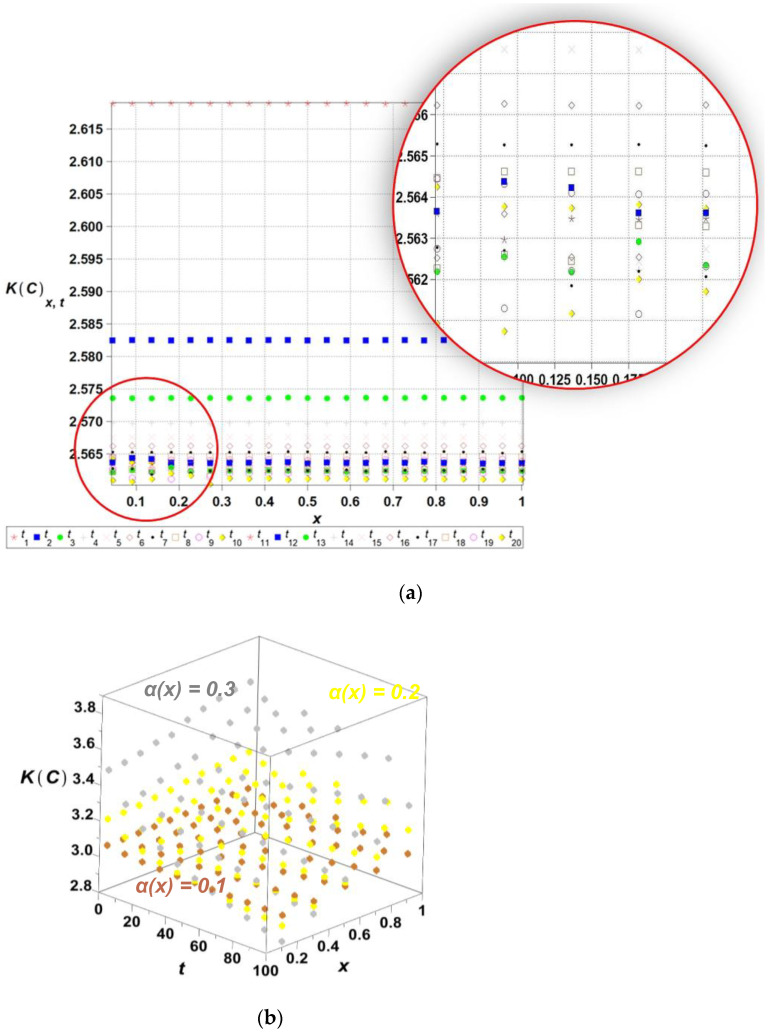
The kurtosis of the concentration *K*(*C*[*x*,*t*]) for selected time $t_{i},i=\{1,2,...,20\}$*,* over the domain xϵ[0,…,1] (**a**) and 3D plot of the kurtosis of the concentration *K*(*C*[*x*,*t*]) for spatially varying diffusion coefficients ∝(x)ϵ{0.1,0.2,0.3}, tϵ[0,…,100] (**b**).

The algorithm proposed has been implemented in the computer algebra environment MAPLE 2025. The length of the channel l has been adopted as the input Gaussian parameter in this problem, where its initial expected value equals $E\left[l_{0}\right]=1.0$, while the coefficient of variation *α*(*l*) takes 0.1. The time series is represented as the linear polynomial, such that $l\left(x,\omega ,t\right)=l_{0}\left(x,\omega \right)+\hat{l}\left(\omega \right)\;t;\;t\in \left[0,20\right]$, where $E\left[\hat{l}\left(\omega \right)\;\right]=0.01$. Figure 3, Figure 4, Figure 5, Figure 6 and Figure 7 contain the results of the determination of probabilistic moments and coefficients for the concentration ratio $C\left(x,\omega ,t\right)$ using the stochastic perturbation-based finite difference method. We have in turn: the expectations (Figure 3), the coefficients of variation (Figure 4), the Shannon entropy (Figure 5) and skewness (Figure 6), and finally, kurtosis (Figure 7). We discretize the entire computational domain, xϵ[0.0,1.0], into 20 equidistant subintervals along its length.

Expected values remain the same for *x* = 0 independently of the time of a process and of the input coefficient of variation, and are equal to 1. On the opposite side of a computational domain, the concentration’s expectation decreases linearly from the initial unit value to 2, also independently of the input coefficient of random dispersion. Spatial variations of the expected value of the concentration are highly nonlinear along a computational domain showing definite convexity independent of a time. The standard deviation of this concentration coefficient is highly dependent upon the input coefficient of the variation—the higher $\alpha \left(l\right)$, the larger the resulting $\sigma \left(C\right)$. They have zero values at any time for x=0, where we apply the deterministic boundary condition, and then they increase moderately to reach the maximum at the opposite edge of a domain. They also increase almost linearly together with time in this particular process.

The Shannon entropy, shown in Figure 5, decreases over time, indicating a progressive reduction in system uncertainty and convergence towards equilibrium. Simultaneously, the coefficients of variation of concentration, shown in Figure 4, exhibit significant fluctuations in early diffusion stages, followed by stabilization, suggesting that initial randomness dissipates as the system evolves. The presented approach offers a refined perspective on diffusion dynamics, with potential applications in reliability assessment, engineering simulations, and environmental modeling. The resulting diagram of the expected concentration profile, along with the corresponding standard deviation (Figure 4b), shows the output coefficients of variation. It is remarkable that for any combination of the parameters of time t and length l, the output coefficient of variation is significantly higher than the input one. The resulting ratio of output versus input coefficients increases together with an input random dispersion level. Finally, one may notice that the output coefficient $\alpha \left(C\right)$ increases nonlinearly together with *l*, and almost linearly with respect to the time t.

The third central probabilistic moments given in Figure 6 are positive everywhere and they also significantly increase together with an input coefficient $\alpha \left(l\right)$. Absolute values of the third moment are very close to 0, while skewness (in Figure 6a) belongs to the interval $\beta \in \left[0.0,1.2\right]$, which gives an almost symmetrical probability density function, especially for smaller values of the input $\alpha \left(l\right)$. Both quantities are equal to *0* for x=0, where the deterministic boundary conditions are applied and, of course, they increase together with *l* and with time t. All the aforementioned surfaces representing parametric variability of the probabilistic moments are very smooth without any local oscillations and singularities, that may result from higher-order partial derivatives.

Finally, we study the fourth central probabilistic moments of the concentration as well as the kurtosis (Figure 7) for three different values of $\alpha \left(l\right)\in \left\{0.10,0.20,0.30\right\}$. Fourth moments appear to be positive, although even smaller than the third ones, while the resulting kurtosis keeps close to 3, especially for $\alpha \left(l\right)=0.10.$ The surfaces obtained for larger values of the input $\alpha \left(l\right)$ are not as regular and smooth as those of the lower probabilistic moments and the coefficients. Their values both increase remarkably, together with the time of a process, but are almost independent of the parameter l. A comparison of Figure 4, Figure 6 and Figure 7 shows that higher probabilistic moments become less and less dependent upon the domain length l.

However, the most important conclusion here is that for smaller random dispersion of the coefficients in the time series representation of $l\left(\omega ,t\right),$ the final concentration factor keeps close to the Gaussian distribution. Its distance to this PDF increases together with the value of the input coefficient of variation $\alpha \left(l\right)$.

## 6. Concluding Remarks

I. As documented above, Shannon entropy may serve as some additional uncertainty quantification and propagation measure close to the coefficients of variation not only in steady-state but also in non-stationary problems with random coefficients. It should be noted that the analysis of coefficients of variation provides precise information about the nature of the process at a given time point, enabling the assessment of the relative dispersion of random values at specific spatiotemporal locations. In contrast, Shannon entropy can serve as a complementary measure for uncertainty quantification and propagation, offering a more global insight into the process dynamics. Unlike the local characteristics described by coefficients of variation, entropy captures the trend of uncertainty propagation over time and space, both in steady-state and non-stationary problems involving random coefficients. As such, it represents a valuable tool for the comprehensive analysis of complex processes in which the distribution of uncertainty evolves significantly over the course of the system’s development. Although the determination of Shannon entropy demands the application of the Monte Carlo simulation, it gives accumulated knowledge about uncertainty propagation, which in probabilistic moments-based analysis follows the graphs of expectations and second-order statistics. It has also been shown that an application of the symbolic algebra environment enables a solution of the generalized stochastic-perturbation-based discrete equations’ system as well as for an introduction of the perturbation parameter ε into the final solution expansions and the resulting probabilistic moments. Thanks to the very extensive linear algebra tools in MAPLE 2025 (as well as in other symbolic computation systems), we can efficiently implement the stochastic perturbation scheme using the response function method [27]. This approach relies on constructing low-order polynomial approximations of the system response with respect to uncertain input parameters and enables analytical computation of output probabilistic moments. As a result, we obtain a compact and flexible framework that is compatible with both symmetric and non-symmetric coefficient matrices, and capable of handling nonlinear effects due to input uncertainty.
II. The numerical analysis presented here demonstrates that Shannon entropy systematically decreases over time, indicating a progressive reduction in uncertainty and increased spatial uniformity of the concentration field. The highest entropy values are observed near the boundary, suggesting localized disorder at the system’s edge. Simultaneously, the coefficient of variation stabilizes rapidly, with initially elevated values diminishing significantly as the system evolves. This convergence highlights the decreasing influence of stochastic fluctuations and the growing regularity of the solution. The consistency between entropy and variance-based indicators confirms that Shannon entropy serves as a reliable measure of uncertainty and information content in stochastic diffusion–reaction processes. These findings support the utility of entropy-based diagnostics in identifying spatial zones of increased randomness and guiding the analysis of complex transport phenomena. Numerical results presented above also indicate that the system evolves towards a steady state, where concentration differences are minimized, and both entropy and the coefficient of variation decrease over time. The initially high entropy and variance highlight the presence of dynamic mixing processes and stochastic fluctuations, which gradually diminish, leading to a more deterministic concentration distribution. These findings confirm that the stochastic modeling approach effectively captures the initial uncertainty within the system and its subsequent reduction as diffusion progresses, providing a robust framework for analyzing reaction–diffusion dynamics in uncertain environments.
III. The generalized stochastic finite difference method seems to be the efficient probabilistic computational tool to model the reaction–diffusion problems with random parameters and time series and should be further developed for unsteady problems, and also for inhomogeneous domains [32] in 2D or 3D [29]. As was demonstrated above, this method can be applied to problems with physical (mechanical or chemical) parameters having some stochastic boundary waviness, although essentially more complicated situations with multiple randomness sources may also be modeled. It has been shown that the stochastic methodology described above may also be applied directly to the implementations of the FDM on irregular grids [33] or combined with some meshless techniques [34]. Further comparative studies against the Monte Carlo simulation programs may give a direct answer concerning the precision of the perturbation-based SFDM in problems that have some input parameters defined as time series with random coefficients.
IV. Although the proposed stochastic finite difference method (SFDM) shows promising results for linear and stationary reaction–diffusion problems, its extension to more realistic, nonlinear, and time-dependent systems remains a significant challenge. In such cases, several numerical difficulties must be addressed, i.e., nonlinearities often introduce stiffness and sensitivity to perturbations, which can severely constrain the choice of discretization parameters. Similarly, time-dependent problems impose additional stability conditions on the numerical scheme, resulting in high computational costs for long-time simulations. To overcome these challenges, future versions of the SFDM should incorporate implicit integration techniques, which are known to offer superior stability properties for stiff systems. Furthermore, adaptive time-stepping strategies could be employed to dynamically control the temporal resolution depending on the rate of change in the system. These enhancements, while increasing computational complexity, would allow the method to handle more general classes of reaction–diffusion systems, including those with strong nonlinearity and spatial heterogeneity.

Work is currently underway to investigate such extensions, with the aim of broadening the applicability of SFDM beyond textbook examples. (See Table 2 and Table 3).

## Figures and Tables

**Table 1:** Computational algorithm for SFDM and entropy determination.

$\mathrm{read}\;\;\;\;\;\;\;\;\;\;\;\;\;\;\;\;\;\;\;\;\;\;\;\;\;\;\;\;\;\;\;\;\;\;\;\;\;\;\;E\left[A_{i}\right]$$,\;Var\left(A_{i}\right)$, *Δt*
for *i* = 1 to *n* do $\;\;\;\;\;\;\;\;\;\;\;\;\;\;\;\;t_{i}=t_{0}+\Delta t$ compute $E\left[l\left(t_{i}\right)\right],\;\;\;Var\left(l\left(t_{i}\right)\right)$ for *m* = 0 to *N* do compute $\sum_{k=0}^{m}\left(\begin{array}{c} m\\ k \end{array}\right)\frac{\partial^{k}K\left(t_{i}\right)}{\partial b^{k}}\frac{\partial^{m-k}C\left(t_{i}\right)}{\partial b^{m-k}}=\frac{\partial^{m}F}{\partial b^{m}}$ for *k* = 0 to *4* do compute $\mu_{k}\left(C\left(t_{i}\right)\right)$ estimate $pdf\{C\left(t_{i}\right)\}$, $h\left(C\left(t_{i}\right)\right)$ end do end do end do

**Table 2:** Symbols and notation.

Symbol	Meaning
*x*	Spatial coordinate (position)
*t*	Time variable
*ω*	Element of the probability space
*ℓ*	Total length of the diffusion domain
*ℓ*(*⋅*)	Uncertain input function or parameter, modelled as random variable
*C*(*x*,*ω*) *or C*(*x*,*ω*,*t*)	Concentration as a function of space, randomness, and time
*C_i_*	Approximation of concentration at the *ith* node
*Δ*	Grid spacing in the finite difference scheme
*C_0_*, *C_1_*	Boundary concentrations at *x = 0* and *x = ℓ*
*b*(*ω*)	Random parameter (e.g., uncertain channel length)
*δ*	Histogram bin width used for entropy calculation
*ε*	Perturbation parameter used in Taylor series expansion
*E*[*⋅ *]	Expected value
*Var*[*⋅ *]	Variance
*CoV*(*⋅*,*⋅*) *or α*(*⋅*)	Coefficient of variation
*StD*(*⋅*,*⋅*)	Standard deviation
*A*(*⋅*,*⋅*)	*3rd* probabilistic moment—skewness
*K*(*⋅*,*⋅*)	*4th* probabilistic moment—kurtosis
*R*(*⋅*,*⋅*)	*Uncentered autocovariance function of a stochastic process X_t_*
*μ_m_*(*⋅*,*⋅*)	*mth* probabilistic moment
*h*	Shannon entropy
*p*(*b*)	The probability density function of the random variable *b*
*A*	A Gaussian random variable used in the time series
SFDM	Stochastic finite difference method
RFM	Response function method
MAPLE	Symbolic computing system used for implementing the algorithm

**Table 3:** Computational list of steps for SFDM and entropy determination.

1. Input: *E*(*l*), *α*(*l*), *Δt*, total time steps *n*, number of moments *m* = 4
2. For *i* = 1 to *n*: a. Compute current time *t_i_* = *t_0_* + (*i–*1) *· Δt* b. For each stochastic coefficient *A_m_*(*t_i_*), compute: i. Expectation *E*[*A_m_*] ii. Variance *Var*[*A_m_*] c. Solve boundary value problem via SFDM d. For *k* = 0 to *m*: i. Compute central moment *μ_k_*(*C*(*x,t_i_*)) ii. Estimate histogram of output variable *b* iii. Compute entropy *h*(*C*(*x,t_i_*)) using histogram probabilities

## Data Availability

The original contributions presented in this study are included in the article. Further inquiries can be directed to the corresponding author.

## References

[1] Collatz L.C. (1960). The Numerical Treatment of Differential Equations.

[2] Forsythe G.E., Wasow W.R. (2013). Finite Difference Methods for Partial Differential Equations.

[3] Minkowycz W.J. (1988). Handbook of Numerical Heat Transfer.

[4] Taflove A. (1998). Advances in Computational Electrodynamics: The Finite Difference Time Domain Method.

[5] LeVeque R.J. (2007). Finite Difference Methods for Ordinary and Partial Differential Equations.

[6] Kleiber M., Hien T.D. (1992). The Stochastic Finite Element Method.

[7] Kamiński M. (2001). Stochastic perturbation approach in vibration analysis using finite difference method. J. Sound Vib..

[8] Kamiński M. (2007). Generalized perturbation-based stochastic finite element method in elastostatics. Comput. Struct..

[9] Ghanem R.G., Spanos P.D. (1991). Stochastic Finite Elements: A Spectral Approach.

[10] Hurtado J.E., Barbat A.H. (1998). Monte Carlo techniques in computational stochastic mechanics. Arch. Comput. Methods Eng..

[11] Jensen H.A., Jerez D.J., Figueroa C. (2025). On the use of reliability methods and Hamiltonian Monte Carlo for complex identification problems in structural dynamics. Mech. Syst. Signal Process..

[12] Prieto F.U., Muñoz J.J.B., Corvinos L.G. (2011). Application of the generalized finite difference method to solve the advection-diffusion equation. J. Comput. Appl. Math..

[13] Schnakenberg J. (1987). A reaction-diffusion problem in the biophysics of photoreceptors. Z. Für Phys. B Condens. Matter.

[14] Shannon C.E. (1948). A mathematical theory of computation. Bell Syst. Tech. J..

[15] Wang Y., Wang X., Cao G. (2025). Information entropy regularization method for structural identification with large-scale damaged parameters. Comput. Methods Appl. Mech. Eng..

[16] Tehseen N., Broadbridge P. (2012). Fourth Order Diffusion Equations with Increasing Entropy. Entropy.

[17] Cincotta P.M., Giordano C.M. (2023). Estimation of diffusion time with the Shannon entropy approach. Phys. Rev. E.

[18] Cincotta P.M., Giordano C.M., Silva R.A., Beaugé C. (2021). Shannon entropy diffusion estimates: Sensitivity on the parameters of the method. Celest. Mech. Dyn. Astr..

[19] Beck C. (2009). Generalized information and entropy measures in physics. Contemp. Phys..

[20] Jüngel A. (2016). Introduction. Entropy Methods for Diffusive Partial Differential Equations.

[21] Desvillettes L., Fellner K. (2008). Entropy methods for reaction-diffusion equations: Slowly growing a-priori bounds. Rev. Mat. Iberoam..

[22] Blanchet A., Bonforte M., Dolbeault J., Grillo G., Vázquez J.L. (2009). Asymptotics of the Fast Diffusion Equation via Entropy Estimates. Arch. Rational. Mech. Anal..

[23] Fischer J. (2017). Weak–strong uniqueness of solutions to entropy-dissipating reaction-diffusion equations. Nonlinear Anal..

[24] Kamiński M. (2021). On Shannon entropy computations in selected plasticity problems. Int. J. Numer. Methods Eng..

[25] Li X., Essex C., Davison M., Hoffmann K.H., Schulzky C. (2003). Fractional Diffusion Irreversibility Entropy. J. Non-Equilib. Thermodyn..

[26] Ingo C., Magin R.L., Parrish T.B. (2014). New Insights into the Fractional Order Diffusion Equation Using Entropy and Kurtosis. Entropy.

[27] Kamiński M. (2013). The Stochastic Perturbation Method for Computational Mechanics.

[28] Ananthaswamy V., Vijayalaskhmi V., Anantha Jothi J.K. (2025). A new approximate analytical method for solving some non-linear boundary value problems in the Reaction-Diffusion model. Comput. Methods Differ. Equ..

[29] Clavero C., Miller J.H., O’Riordan E., Shishkin G.I. (2001). Numerical experiments for advection-diffusion problems in a channel with a 180 bend. Appl. Numer. Math..

[30] Kunert G. (2005). A posteriori H1 error estimation for a singularly perturbed reaction-diffusion problem on anisotropic meshes. IMA J. Numer. Anal..

[31] Cox D.R., Miller H.D. (1970). The Theory of Stochastic Processes.

[32] Yeh C.L. (2004). A singularly perturbed reaction-diffusion problem with an inhomogeneous environment. J. Comput. Appl. Math..

[33] Liszka T., Orkisz J. (1980). The finite difference method at arbitrary irregular grids and its applications in applied mechanics. Comput. Struct..

[34] Mirzaee F., Samadyar N. (2020). Combination of finite difference method and meshless method based on radial basis functions to solve fractional stochastic advection-diffusion equations. Eng. Comput..

